# Effect of Palpable Udder Defects on Milk Yield, Somatic Cell Count, and Milk Composition in Non-Dairy Ewes

**DOI:** 10.3390/ani11102831

**Published:** 2021-09-28

**Authors:** Mandefrot M. Zeleke, Paul R. Kenyon, Kate J. Flay, Danielle Aberdein, Sarah J. Pain, Sam W. Peterson, Anne L. Ridler

**Affiliations:** 1 School of Veterinary Science, Massey University, Private Bag 11222, 4410 Palmerston North, New Zealand; D.Aberdein@massey.ac.nz (D.A.); a.l.ridler@massey.ac.nz (A.L.R.); 2 School of Veterinary Medicine, Wolaita Sodo University, Wolaita Sodo P.O. Box 138, Ethiopia; 3 School of Agriculture and Environment, Massey University, Private Bag 11222, 4410 Palmerston North, New Zealand; p.r.kenyon@massey.ac.nz (P.R.K.); S.J.Pain@massey.ac.nz (S.J.P.); S.Peterson@massey.ac.nz (S.W.P.); 4 Department of Veterinary Clinical Sciences, City University of Hong Kong, Kowloon, Hong Kong 999077, China; kateflay@cityu.edu.hk

**Keywords:** hard udder, lactation, milk composition, milk yield, somatic cell count, udder health

## Abstract

**Simple Summary:**

The occurrence of udder defects in ewes impacts the productive lifespan of the affected ewe and reduces their lamb production. This study investigated the effects of palpable udder defects on milk yield and milk composition in Romney ewes. The findings showed that the effect of udder-half defects on udder-half milk yield was substantial; however, there was no difference in individual (whole udder) ewe milk production between ewes with one udder-half defective and both normal udder-halves. This was due to a compensatory increase in milk production of the normal udder half when the other udder half was defective, with the exception being ewes that retained the defects for several weeks. No notable difference in milk composition was observed between defective and normal udder halves, except for solids non-fat (SNF). This study shows that udder defects affect milk production in non-dairy ewes, highlighting the potential effects on lamb production.

**Abstract:**

In non-dairy ewes, udder defects hinder the survival and weight gain of their pre-weaned lambs. The objectives of this study were to determine the effects of palpable udder defects on milk yield, somatic cell count (SCC), and milk composition in non-dairy Romney ewes. Ewes with a history of udder defects or normal udders were selected for the study. Of a total of 48 ewes that lambed, 30 ewes reared at least one lamb, and were milked six times, once weekly, for the first six weeks of lactation. Udder halves were palpated and scored at each milking event. Multivariate linear mixed models examined the impacts of udder defects on udder-half and whole-udder milk yield, SCC, and milk composition (fat, protein, lactose, total solids, and solids non-fat (SNF)). Across the six examinations, 24.7% of the total 352 udder-half examinations were observed to be defective. Udder halves that were defective at least once produced on average 57.9% less (*p* < 0.05) milk than normal udder halves, while normal udder halves with a contralateral defective half yielded 33.5% more (*p* < 0.05) milk than normal udder halves. Successive occurrence of both hard and lump udder defect categories in an udder-half, udder defect detection early in lactation, and a high frequency of udder defect detection were all associated with udder-half milk yield loss (*p* < 0.05). At the whole-udder level, no differences in milk yield (*p* > 0.05) were observed between those with one udder-half defective and both normal udder-halves. However, udders in which one udder half was categorised as hard but progressed to lump and remained as lump until 42 days of lactation produced less (*p* < 0.05) milk compared with normal udders. With the exception of SNF, there were no significant associations (*p* > 0.05) between milk composition parameters and udder defect. Overall, these findings emphasise the importance of udder health in non-dairy ewes and the potential effect of udder defects on their lambs.

## 1. Introduction

Various udder defects including mastitis have been reported in both dairy and non-dairy ewes [1,2]. Clinical presentations of these defects include signs of inflammation, abnormal secretion, abscesses, lumps, nodules, diffuse hardness, and cysts [1,2,3]. Udder defects cause mild to serious damage to the mammary gland in ewes [4,5,6]. This damage can hinder the synthetic capacity of epithelial cells and/or increase the permeability of blood components into the milk [7,8]. The occurrence and impact of these defects varies greatly depending on many factors, such as the individual animal, causative agent, severity of infection, management factors, production systems, genetics/breed, udder morphology, production level, and whether both or single udder-halves are involved [9,10,11]. Globally, the prevalence of udder defects in non-dairy ewes ranges from 0 to 10.9% [2,12,13,14,15,16]. The prevalence of udder defects in New Zealand is up to 7% [3,17,18,19,20,21].

Udder defects in dairy ewes can be detected more frequently compared to non-dairy ewes because farmers check the udder during regular daily milking events [22]. In non-dairy ewes, udder defects are usually only identified at weaning or prior to mating, when farmers are selecting ewes for the next breeding season [2]. However, a condition described as “hard udder” has been observed during early lactation [23], while the prevalence of clinical mastitis has been reported to be higher shortly after lambing [24], which is a time when most farmers would not be checking the udders of non-dairy breed ewes in outdoor lambing systems.

Clinical and sub-clinical mastitis have a significantly negative impact on milk yield in ewes [4,24,25,26,27]. In dairy ewes, studies on the effect of mastitis on milk yield and composition have been undertaken for the purposes of quantifying milk losses and assessing milk quality for cheese production [7,28]. In contrast, in non-dairy ewes, the focus of studies investigating mastitis [14,27,29] or palpable udder defects [1,15,18] have been to investigate growth rates and survival of pre-weaned lambs. Mastitis decreases milk yield and alters milk composition, including protein, fat, and lactose [7,18,30]. Lactose concentration decreases in mastitic (subclinical and clinical) ewes because of tissue damage, which interferes with its synthesis [28] and osmotic regulation role, which accentuates the decrease [27]. Changes in milk fat and protein levels vary depending on the volume of milk loss or the dilution effect during mastitis [31,32].

An individual ewe whole-udder assessment approach has been used to diagnose mastitis, quantify milk yield loss, and assess milk composition for both dairy and non-dairy ewes [5,28,33]. However, due to the anatomic, functional, and infection independency of the two udder-halves, and the compensatory effects in both unilateral and bilateral infection, assessment of the whole-udder has been associated with huge individual variation [11,25,34]. Hence, determining milk losses due to mammary infection is better studied by assessment of individual udder-halves [27].

In non-dairy ewes, there are a limited number of studies on the impact of udder defects on milk yield despite the potential serious consequences to lamb growth and survival to weaning and subsequent culling of affected ewes [1,15,18]. “Hard udder”, a diffuse hard consistency of the udder occurring at or shortly after parturition, has been reported to result in udders that appear to be full of milk but fail to express milk after lambing [20,23]. Hard udders have been associated with teat cord (hard core) formation, which may prevent milk removal [19]. Ewes whose mammary glands contain lumps have been associated with decreased body weight gain in pre-weaned lambs [35]. The presence of mammary glands with lumps is also a reported reason for culling of ewes, as it is assumed they have decreased milk production [15,19] and reduced overall productivity [36]. Moreover, milk samples collected from ewe udders with defects including nodules, lumps, diffuse hardness, and abscesses were reported to contain clots and flakes, serous and purulent fluid, and blood-stained secretions [2].

To the authors’ knowledge, no previous studies have assessed the effect of palpable udder defects on milk yield and composition in either dairy or non-dairy ewes. Therefore, the objectives of this manuscript were to quantify the effects of palpable udder defects on udder-half and whole-udder milk yield, somatic cell count, and milk composition in mixed-age non-dairy breed (Romney) ewes. It was hypothesized that udders with palpable defects would show decreased milk yield and altered milk composition compared with normal udders.

## 2. Materials and Methods

### 2.1. Ewe Selection

Eighty commercial ewes aged five to six years were selected from Massey University’s Riverside farm, Wairarapa, New Zealand, based on udder and teat traits evaluated during the previous three years (2016, 2017, and 2018) and at the time of selection [18]. In total, 60 ewes with defects or a history of defects and 20 with consistently normal udders were selected. These ewes were transported to Massey University’s Keeble farm (Palmerston North, New Zealand) in January 2019.

### 2.2. Animal Management

From the time of arrival at Keeble farm, the ewes were managed as a single flock under commercial grazing conditions. For breeding, ewes were progesterone-synchronised by inserting a controlled release intravaginal device (Eazi-Breed CIDR^®^, Zoetis New Zealand) on 13 March 2019 for a period of 12 days. Pregnancy diagnosis was undertaken twice in mid-pregnancy (4 June and 25 July) using trans-abdominal ultrasonography undertaken by a commercial operator to confirm pregnancy and foetus numbers. Single and twin-bearing ewes were retained while non-pregnant and triplet-bearing ewes were culled (*n* = 21).

On 30 July 2019, all ewes were weighed, body condition scored, and moved to a single paddock closer to the milking parlour with a pasture requirement according to commercial conditions for late pregnant ewes (pre-lambing). Forty-eight ewes were retained for lambing and the lambing period occurred between 13 August and 9 September 2019. On milking days (see below), lambs were separated from their dams for six hours between morning and afternoon milkings, during which time each lamb was bottle-fed 300 g of ewe’s milk [37]. If a ewe produced less than 1200 g/day of milk or abandoned their lamb/s, the lamb/s was/were removed from the ewe and fostered. These ewes, and ewes for whom all their lambs died (18), were excluded from the milking study.

### 2.3. Milking and Milk Yield Calculation

Each ewe was milked six times during lactation at seven-day intervals, beginning 5–8 days after lambing. There were two milking groups: ewes that lambed on Sunday-Tuesday were milked on Mondays while those that lambed on Thursday-Saturday were milked on Fridays. Ewes that lambed on Wednesday (21 August 2019) were milked once on Wednesday and then joined the Friday milking group to balance group sizes. Each milking group contained ewes with a history of both normal udders and udder defects. The milking procedure was as follows. Briefly, milking was achieved by injecting oxytocin (oxytocin-EA (Ethical Agents Ltd., Auckland, New Zealand) (1 IU) diluted with 0.9 mL of physiological saline into the jugular vein 0.5–1 min before milking commenced [37]. At each milking (morning (approximately starting at 9 am) and afternoon (approximately starting at 3 pm)), ewes were machine-milked for approximately two minutes after which any remaining milk in the mammary gland was expressed by hand. In the morning, the time of milking was recorded individually for each ewe. The ewes, without their lambs, were then put back onto pasture until the afternoon milking. In the afternoon, milk from each udder-half was collected into a separate container and the weight of milk (g) collected and time were recorded. To estimate a ‘daily’ milk yield, the weight of milk collected from the afternoon milking and the inter-milking interval time were used to calculate a ‘daily’ (24 h) milk yield from each udder-half. The ‘daily’ whole-udder milk yield was calculated as the sum of milk yield from two udder halves for each ewe [38]

### 2.4. Milk Sampling and Laboratory Analysis

The milk collected was gently mixed by inverting the collection container and 10 and 50 mL of milk were removed into separate sampling vials for somatic cell count (SCC) and milk composition analysis, respectively. SCC was assessed 2–4 h post-collection on the same day (see details below). Milk samples collected for composition analysis were stored in a refrigerator (+4 °C) overnight and analysed the next day.

### 2.5. Somatic Cell Count (SCC) Determination

Somatic cell count analysis of milk from each udder-half was performed using a DeLaval cell counter (DCC) (DeLaval Int. AB, Tumba, Sweden) according to the method of Gonzalo, et al. [25]. Milk was extracted from the sample vial into the DCC cassette, which contained a small amount of fluorescent stain and Triton X-100, and provided cell counts per ml within approximately 1 min. The DCC captured a digital picture of each somatic cell’s nucleus, which was then stained in the cassette with a DNA-specific fluorescent reagent enabling each cell’s nuclei to be counted. A small number of samples with clots or blood (signs consistent with clinical mastitis) could not be analysed as the presence of clots meant they were unable to be read by the machine; these were excluded from the analysis.

### 2.6. Milk Component Analysis

Analysis of milk components including fat, protein, lactose, total solids, and non-fat solids (NFS) were determined using a Milkoscan FT1 (Foss Electric, Hillerød, Denmark). The stored milk samples were placed in a water bath (40 °C) before the analysis and carefully inverted to homogenize the fat. The Milkoscan was set to allow triplicate analyses from the single sample and provided these three results along with the mean and standard deviation. Sheep milk calibration was developed using Fourier transform infrared spectroscopy (FTIRS) through a Milkoscan FT1 machine and the details of the Milkoscan calibrations are summarized in Table A1.

### 2.7. Udder Scoring

Udder scoring of the ewes was undertaken using the methods described by Griffiths, et al. [18] each week for the first six weeks of lactation (on the same day as milking). All ewes were udder-scored at these times, regardless of whether they reared a lamb and were milked.

### 2.8. Data Management

For analysis of the data, the 7-category udder scoring system as described by Griffiths, et al. [18], was condensed into three categories: Score 7 was categorised as ‘hard’, scores 3–6 as ‘lump’, and scores 1 and 2 as ‘normal’ [18]. Udders or udder halves categorised as ‘hard’ or ‘lump’ were considered defective. During the six examinations in lactation, it was observed that most udder-half defects changed category over time (e.g., hard changed to lump). To account for these changes and potential effects on the trait measured, additional sub-categories were developed (Table 1).

Combined over the six once-weekly milk collections from the 30 ewes, the dataset for udder-half consisted of 352 daily milk yields. Four ewes missed the last milking. When udder-half observations with no milk were excluded, the dataset included 262 values. This was further reduced to 255 values for SCC and 243 values for milk composition analyses. Fewer samples were available for SCC and milk composition due to no expression of milk or an inadequate volume of milk to conduct the appropriate tests. The total number for the analysis of whole-udder milk yield consisted of 176 whole-udder observations (i.e., half of 352 daily yields; however, only 123 individual udder-half observations expressed milk from both udder halves). For the analysis between the overall defect type and whole-udder milk yield, six observations from five udders with bilateral udder defects were excluded (*n* = 170).

### 2.9. Statistical Analysis

All data were entered to MS-Excel (2016) and analysed using the statistical package R version 3.6.0 [39]. Analyses were undertaken both at the udder-half and whole-udder level.

#### 2.9.1. Udder-Half Data Analysis

Udder-half milk yield, milk natural log-SCC, and milk composition (fat, crude protein, lactose, total solids, and SNF) were the dependent variables considered. Independent variables considered included udder-half defect occurrence, number of lambs born, number of lambs reared, udder-half (left/right), pre-lambing body condition score (BCS), pre-lambing body weight, udder defect history (over the previous three years), age of ewe, and days in milk. SCC was considered as a predictor variable of milk yield and composition only in those udder halves that expressed milk.

The association of udder-half milk yield was also analysed with various udder-half defect parameters (Table 1). The association of some udder-half defect variables (weekly defect occurrence and weekly and overall udder-half defect type) with SCC and milk composition were not undertaken due to the low numbers of defective udder halves from which milk could be expressed and tested for SCC and composition.

#### 2.9.2. Whole-Udder Data Analysis

The association between whole-udder milk yield and udder defect parameters is summarized in Table 1. The same independent variables were considered as those for the udder-half analysis except for udder-half (left/right).

### 2.10. Multivariate Model Development and Diagnostics

Multivariate linear mixed models with ewe identification number as a random effect and AR1 correlation structure were used to analyse the impact of udder-half or whole-udder defect occurrence, types, frequency, and first detection time, on milk yield, natural log-SCC, and milk composition. For this purpose, “nlme” package in R was used. A stepwise variable selection based on significance level (*p* < 0.1) was implemented. Interaction of days in lactation with udder defects and its quadratic relationship was tested during the models’ development. The confounding effect of variables was progressively assessed during steps of model development by monitoring for a substantial increase in beta coefficient and/or decrease in standard errors.

Histograms and QQ plots were used assessing the normality of the data. Homoscedasticity and linearity were checked using fitted vs. predicted values plot, and the variance inflation factor (VIF) was used to determine the multicollinearity among independent variables. R^2^ was used to assess the goodness fit for the models. Outlier observations were removed based on Cook’s distance and improvement of R^2^. Least squares mean (LSM ± SEM) of dependent variables were calculated by adjusting for significant fixed-effect variables.

## 3. Results

### 3.1. The Occurrence of Udder-Half Defects (Weekly and Overall)

From the total of 48 ewes that lambed, 18 ewes were excluded from the milking study in the first week of lactation because they had lost all their lamb/s. Based on udder palpation seven days after lambing, of these 18 ewes, 39% were categorised as hard in both udder halves, 33% were categorised as hard in one udder-half, and 28% were categorised as lump in one udder-half. Of the 30 ewes that were milked, 14 were single bearing and 16 were twin-bearing, with 9 twin-bearing ewes rearing both lambs.

**Figure 1:**
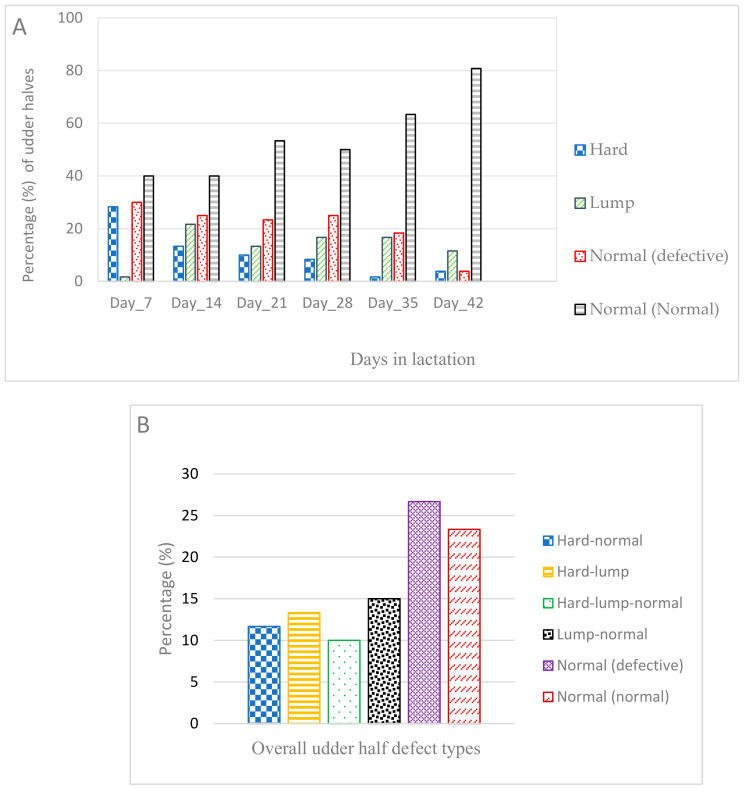
The percentage (%) of udder-half defect occurrence (*n* = 60, i.e., 30 ewes in total), by category in non-dairy ewes whose udder was scored once weekly for the first six-weeks of lactation. (**A**): percentage of weekly udder-half defect types; (**B**): Percentage of overall (i.e., classification over the entire six measurements) udder-half defect types; normal udder halves (both in weekly and overall) were sub-categorised into normal (normal) and normal (defective) to account for possible compensatory effects of a normal udder-half when the contralateral udder-half was defective. Normal (normal) refers to a normal udder-half from a ewe in which both udder halves were normal. Normal (defective) refers to a normal udder-half from a ewe where the contra-lateral udder-half was defective. The naming of the overall (progressive) defect categories describes the change in the defect category over time (e.g., hard-lump refers to an udder-half that was first categorised as ‘hard’ then subsequently was categorised as ‘lump’ and remained ‘lump’ until day 42. Note: Four ewes did not have an udder examination on day 42.

During the udder palpation assessments, it was observed that most ewes’ udder-half defect category changed over time. Figure 1 summarises the occurrence of weekly (A) and overall (B) udder-half defects during the six-week period. The maximum number of udder-half defects was recorded on day 21 (week 3, 35% of 60 udder halves) and the minimum on day 42 (week 6, 5% of 52 udder halves, Figure 1). Across all six examinations, 87 udder halves (24.7% of the 352 udder-half observations) were defective. Note that four ewes (eight udder halves) were not milked on day 42.

The highest number of udder halves in the category ‘hard’ occurred at day seven (28% of the 60 udder halves, Figure 1A) but decreased to 3.8% by day 42 (week six). A total of 35% of the 60 udder halves were categorised as ‘hard’ at least once during the six-weeks. In contrast, udder halves in the category ‘lump’ were lowest at day seven (week one, 1.6% of the 60 udder halves) but had increased to 16.7% by day 14 (week two) and remained at similar levels to day 42 (week six). A total of 35% (of the 60 udder halves) were categorised ‘lump’ at least once over the six weeks. Combined over all six weeks, 50% of 60 udder halves were defective at least once (Figure 1B). Most of the udder-half defects (83.3% of the 30 defective udder halves) were detected in the first two weeks (14 days) post-lambing. Five udder halves (16.7% of the 30 udder halves) were identified as defective only once, while the rest were observed to be defective on two or more occasions.

During the six weeks, many of the udder-half defects changed in category over time. A third of the 21 udder halves initially categorised as ‘hard’ changed to normal and the rest changed to ‘lump’ (Figure 1B). Of those that changed to ‘lump’, more than half remained as ‘lump’ while the others changed to normal.

### 3.2. Udder-Half Milk Expression

Based on weekly milking status, 18/60 (30%) of the udder halves included in the study expressed no milk at least once during the six-week period. At day 7, 12/60 (20% of the udder halves) expressed no milk and this increased to 16/60 (27%) on day 35 (i.e., week five). Of these 18 udder halves that expressed no milk, 55.5% expressed no milk on all six occasions, 16.6% on five, 22.2% on four, and 0. 5% on just one occasion.

Over the entire 42-day (six-week) milking period, data were available for 352 udder halves of which 90 had no milk expression. Of these 90 udder halves, 28% were in the category ‘hard’, 38% were ‘lump’, while 34% were ‘normal’. In contrast, when an individual half udder was categorised based on the entire six-week (42-day) period (category 2 in Table 1), the 30 normal (normal) and normal (defective) udder halves expressed milk at every milking. Of the 30 udder halves that expressed no milk, 23% were categorised as hard-normal 27% hard-lump, 20% hard-lump-normal, and 7% lump-normal.

### 3.3. Relationship between Udder-Half Defect Occurrence and Udder-Half Milk Yield

**Figure 2:**
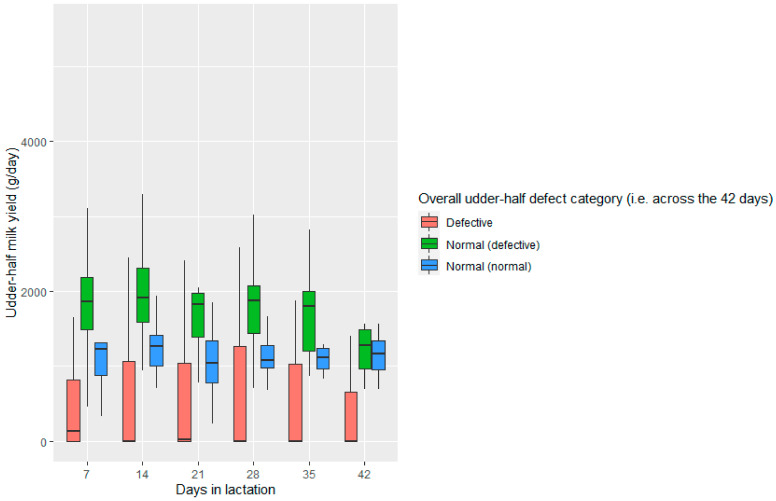
Udder-half milk yield (median and interquartile range) based on overall udder-half defect (i.e., across the 42 days/six-week period) categories in non-dairy Romney ewes (*n* = 30) milked once weekly during the first six weeks of lactation. Udder halves that were categorised as ‘hard’ or ‘lump’ on at least one of the six occasions were considered Defective. Normal (defective): was used to describe a normal udder-half where the contralateral udder-half was defective. Normal (normal): was used to describe a normal udder-half where the contralateral udder-half was also normal.

Normal (normal) udder halves produced more (*p* < 0.01) milk than defective udder halves based on both weekly and overall udder-half defect analysis (Table 2 and Figure 2). Further, normal (defective) udder halves produced more (*p* < 0.01) milk than normal (normal) udder halves. Overall, defective udder halves (defective at least once during the six-weeks) produced 57.9% less milk than normal (normal) udder halves. In contrast, normal (defective) udder halves produced 33.5% more milk than normal (normal) udder halves.

Using the multivariate model, weekly udder-half defect occurrence and the number of lambs each ewe was rearing (rearing rank) were predictors (*p* < 0.05) of udder-half milk yield (R^2^ = 0.366, Table 3), where weekly udder-half defect occurrence explained 36.6% of the variation in milk yield. The overall udder-half defect explained 39% (*p* < 0.05) of the milk yield at the half udder level (Table 3).

Further analysis was undertaken to assess the relationship between udder-half defect occurrence and udder-half milk yield, using only those udder halves where milk was actually expressed (*n* = 262, of the total 352 potential udder-half collections over the six-week period). This analysis identified differences (*p* < 0.05) among weekly and overall udder-half defect categories; however, overall, the differences between the normal and defective categories were less than those identified in the analysis including all 352 udder halves as outlined earlier (Table 2 and Table 3).

### 3.4. Relationship between Udder-Half Defect Occurrence and Somatic Cell Count (SCC)

The log-SCC was not affected (*p* > 0.05) by overall udder-half defect occurrence (Table 2). Among the independent variables included for udder-half log-SCC analysis, the best fit for the multivariate linear mixed model included birth rank and udder defect history as predictors (*p* < 0.05, R^2^ = 0.46, Table 3), where ewes that gave birth to twins and those with a previous history of udder defects had higher log-SCC than those that gave birth to a single lamb and had no history of udder defects.

### 3.5. Relationship between Udder-Half Defect Occurrence and Milk Composition

There was no association between overall udder-half defect occurrence and milk fat, crude protein, total solids, and lactose (*p* > 0.05, Table 2). However, defective udder halves had lower (*p* < 0.05) SNF than the normal (normal) udder halves, whereas no difference was observed between normal (defective) and normal (normal) udder halves (*p* > 0.05).

Days in lactation and SCC were significantly correlated with milk fat, lactose, and SNF (*p* < 0.05, Table 3). As days in lactation progressed, fat and total solids increased, but SNF decreased (*p* < 0.05). Fat percentage increased with SCC whereas lactose and SNF were negatively associated (*p* < 0.05).

### 3.6. Relationship between Udder-Half Defect Type and Udder-Half Milk Yield

Udder halves categorised as hard or lump at each examination had lower milk yield (*p* < 0.05) compared with normal (normal) udder halves (Table 4). There were no udder-half milk yield differences (*p* > 0.05) in the overall udder-half defect category (hard-normal) compared with normal (normal) udder halves. However, udder halves that were categorised as hard and then progressed to lump and either remained as lump (hard-lump) or progressed to normal (hard-lump-normal) had a lower milk yield than normal (normal) udder halves (Table 4). Additionally, milk yield in udder halves identified as lump that changed to normal (lump-normal) was lower (*p* < 0.05) than normal (normal) udder halves (Table 4). For the multivariate analysis of udder-half weekly milk yield, there was a significant interaction (*p* < 0.05) between days in lactation and udder halves categorised as hard (Table 4). This was due to an increase in the udder-half milk yield in the later days of lactation for udder halves categorised as hard (*p* < 0.05). This is likely explained by a decreased occurrence of udder halves categorised as hard as the days in lactation advanced, as the category changed to lump or normal (Figure 1). Hard-lump and hard-lump-normal udder-half categories had lower (*p* < 0.05) udder-half milk yield compared with normal (normal) udders (Table 4).

### 3.7. Relationship between Udder-Half Defect Frequency and Udder-Half Milk Yield

Udder halves with a high udder defect frequency (defects identified on three to six occasions) produced less milk (*p* < 0.05, 88.5% less) than normal (normal) udder halves (Table 5). However, no differences (*p* > 0.05) were observed between udder halves with low defect frequency (one to two occasions) and normal (normal) udders. The best fit multivariate model for udder-half milk yield consisted only of udder-half defect frequency, which explained 46% (*p* < 0.005) of the variation (Table 5).

### 3.8. Relationship between First Defect Detection Time and Udder-Half Milk Yield

Udder halves detected with a defect for the first time on day seven (end of week one) had lower (*p* < 0.05) milk yield than normal (normal) udder halves, but there was no difference (*p* > 0.05) in milk yield between udder halves detected with a defect on day 14 (end of week two) or later and normal (normal) udder halves (Table 5). The best fit multivariate model for udder-half milk yield consisted only udder-half defect first detection time, which explained 29% of the variation (Table 5).

### 3.9. The Occurrence of Whole-Udder Defects

**Figure 3:**
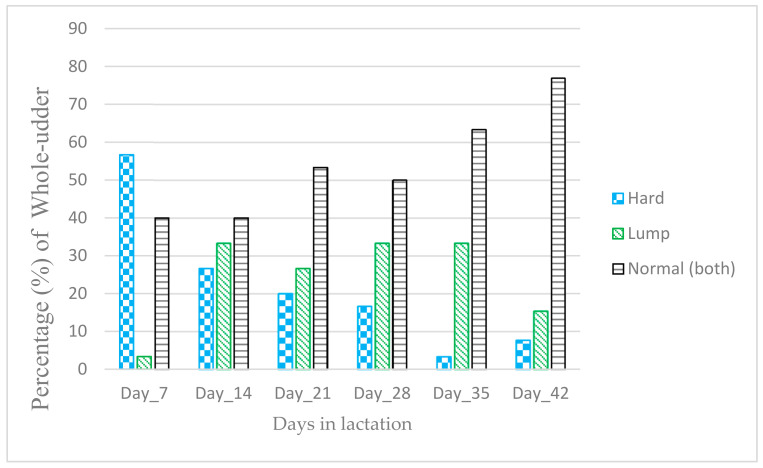
The percentage (%) of weekly whole-udder defect types in 30 non-dairy Romney breed ewes udder scored once weekly during the first six weeks of lactation. The naming of the overall (progressive) whole-udder defect types describes the change in defect category over time: Hard: was used to describe occurrence of ‘hard’ in one udder-half and ‘normal’ or ‘lump’ on the other udder halves; Lump: was used to describe occurrence of ‘lump’ either in one or both udder halves; Normal-both: both udder halves normal; For weekly whole-udder defect, individual udder-half could be identified in a different defect category each week over the six-week (42 days) period. Note four ewes were not udder scored on day 42 (end of week six).

Figure 3 shows the occurrence of weekly whole-udder defects during the first six weeks of lactation. A total of 23 whole-udders from the 30 ewes (77%) were defective at least once during the six once-weekly examinations. Most whole-udder defects were unilateral (i.e., defect in one udder-half only), except in five ewes, in which a bilateral defect was observed on one or two occasions.

### 3.10. Relationship between Whole-Udder Defect Occurrence and Whole-Udder Milk Yield

There was no association (*p* > 0.05) between weekly or overall udder-half defect occurrence and whole-udder milk yield. The best fit regression model had rearing rank as a predictor with an intercept of 1532.8 g/d (±SE 255.70) and a rearing rank coefficient of 410.79 (±SE 185.8). As such, twin-rearing ewes displayed higher (*p* < 0.05) whole-udder milk yields compared to single-rearing ewes. Similarly, there was no difference (*p* > 0.05) in whole-udder milk yield between normal (both) and defective whole-udder categories from those udders (*n* = 123) which expressed milk from both udder halves.

### 3.11. Relationship between Defect Type and Whole-Udder Milk Yield

Weekly whole-udder defect type had no effect (*p* > 0.05) on whole-udder milk yield (Table 6); however, the whole-udders categorised as hard-lump produced less (*p* < 0.05, 20.7% less) milk compared with normal (both) whole-udders. The overall whole-udder defect and rearing rank were predictors (*p* < 0.05) for the best fit multivariate model (R^2^ = 0.29) for whole-udder milk yield (Table 6).

### 3.12. Relationship between Whole-Udder Milk Yield and Whole-Udder Defect Frequency

There were no differences (*p* > 0.05) in whole-udder milk yield between whole-udder defect frequency categories (high: defect identified on three to six occasions, or low: one to two occasions) compared to normal (both) udders (data not shown).

### 3.13. Relationship between Whole-Udder Milk Yield and Whole-Udder Defect First Defect Detection Time

Whole-udder milk yield was not different (*p* > 0.05) between udders where both halves were normal compared with udders where one or both udder halves were found to be defective at either day 7 or day 14 and later (data not shown).

## 4. Discussion

This study was undertaken to assess the effect of palpable udder defects on milk yield and milk composition in non-dairy breed (Romney) ewes. Udder defects and milk parameters were analysed both at the udder-half and whole-udder level. Udder defects are relatively common in ewes in New Zealand, with previous studies undertaken between weaning and mating reporting a 2.3–7% prevalence [3,19,21]. Recently, Griffiths, et al. [18] studied palpable udder defects in Romney ewes at pre-mating, pre-lambing, docking (approximately four weeks post-lambing), and weaning and reported an occurrence of udder defects of 6.0, 5.0, 7.5, and 7.4%, respectively. The occurrence of palpable udder defects in early lactation is not well understood, but it has been suggested to be higher than at other times [19]. Internationally, studies on udder defects in non-dairy ewes have shown comparable occurrence rates to those reported in New Zealand and have impacted mammary health and lamb survival and growth [12,13,14,15,16]. Despite this, there appears to be no available reports on the effects of palpable udder defects on milk yield and milk composition in ewes.

The present study investigated udder defects in a unique population of ewes that had a history of either known udder defects or no defects in the previous three years. From the total 48 ewes that lambed in the present study, 41 ewes (23 of 30 milked ewes and all 18 of the non-milked ewes) had an udder defect in one udder-half on at least one occasion, during their six once-weekly examinations. Thirty eight percent of the ewes that lambed in this study lost all their lambs and a further 12.5% lost one of their twins. These loss rates are higher than the averages reported in single or twin lambs in New Zealand [18,40,41]. In contrast, in the present study, for ewes where both udder halves were categorised as normal throughout the study, all lambs survived. This finding agrees with the potential undesirable impact of udder defects on lamb survival reported previously [1,15,18] and emphasises the importance of udder health and inspection of udders. Ewes that lost all their lambs in the present study either did not express, or only produced very small amounts of, milk from one or both udder halves. Additionally, more than half of the 30 ewes enrolled in the milking study did not express milk from one of the udder halves at least once during the six milkings. Total failure to express milk was seen mainly in udder halves categorised as hard at the time of milking or previously. Similarly, a condition described as hard udder has been reported to interfere with colostrum consumption or cause complete absence of milk, resulting in higher loss rates of lambs and increased culling of ewes [20,23].

Diffuse hardness of the mammary gland (‘hard’) and lumps of various size and consistency (‘lump’) were the only categories of palpable udder defects presented in this study. Repeated weekly udder examinations over the six-week period revealed that the palpable udder defects changed over time from either defective to normal or from one defective category to another. Overall, it was observed that the occurrence of udder defects decreased as lactation advanced, particularly in relation to ‘hard’. The higher occurrence of udder defects observed in the early lactation period could be associated with higher milk production during this time, which may lead to a reduction in immunological competence [42] or a change in the severity of infection [43]. A change from hard to normal palpation scores in the weeks following parturition has been previously reported by others [19,20,23]. However, in some cases, this change took several weeks. This agrees with Grant, et al. [15], who reported that some palpable udder defects persisted during lactation and that these may increase the risk of reoccurrence in the following lactation. This may explain the high rates of udder defects reported in this study, as many of these ewes had a history of udder defects in the previous year/s, with ewes selected specifically because they had a history of palpable udder defects. It might also partially explain the occurrence of a higher percentage of palpable udder defects during lactation than in pregnancy [15,18].

In this study, which included ewes with a history of udder defects, there was a high occurrence of udder halves categorised as hard at day 7, but which subsequently changed to lump or normal within 2–6 weeks post-lambing. Traditionally, farmers in New Zealand examine the udders of ewes at weaning and/or pre-mating. A recent longitudinal cross-sectional study by Griffiths, et al. [18] assessed ewes’ udders during late pregnancy, mid-lactation, weaning, and pre-mating. However, New Zealand-based studies examining udders in early lactation are limited and these included only a small number of ewes and the only focus was a condition described as hard udder [20,23]. Further investigation of udder defects in early lactation could provide further valuable information to explain the pathological process and bacterial flora associated with this defect and the process of progression over time.

Udder halves with a defect observed at least once in six-weekly examinations produced 57.9% (range 27.5 to 99.8%) less milk than udder halves categorised as normal over the entire period. Further, normal udder halves yielded at least twice as much milk compared with udder halves initially categorised as hard but which changed to lump or to lump and then normal over the six-week period (i.e., hard-lump or hard-lump-normal). The loss of milk yield in these udder halves was much higher than those categorised as either hard or lump alone. Additionally, udder halves with a higher udder-half defect detection frequency (i.e., defect detected on more than three out of six examinations) produced up to 88.5% less milk than the normal udders, while udder halves detected with a defect in the first week of lactation produced 65.1% less milk than a normal udder-half. Based on these results, it is apparent that sequential occurrence of both hard and lump (e.g., hard-lump) early in the lactation period, and/or persistency of defects over time, resulted in a greater loss of udder-half milk yield. There are no other studies available that report the effect of palpable udder defects on milk yield. However, the occurrence of sub-clinical mastitis in early lactation has been associated with a significant loss of milk yield in ewes [11,30,44].

In the present study, normal udder halves contralateral to a defective udder half (i.e., described as normal (defective)) produced higher milk yield than normal udder halves with a contralateral normal (i.e., described as normal (normal)). This is most likely due to a compensatory increase in milk production by the normal udder halves in response to the decrease by the contralateral defective. As a result, whole-udder milk yield presented no difference between defective and normal udders, except only in the case of whole-udders detected with hard that changed to lump and persisted (hard–lump). Marti De Olives, et al. [11] quantified a 15–17% loss of whole-udder milk yield in udders detected with unilateral sub-clinical mastitis compared to normal udders. Hence, while there is a level of compensation by the normal udder-half, it may not be enough to compensate fully for reduced production in the impaired udder, which would impact on total lactation yield and lamb growth and survival as was demonstrated in a large field study [18]. In ewes with an udder-half defect or no milk expression from one udder-half, twin lambs would likely be most affected due to competition to access adequate milk yield compared to singletons. Even in a fully functional udder, the milk production of twin-rearing ewes is not twice that of a ewe rearing a singleton [45]. This situation would likely exacerbate the effect of udder defects on twin lamb survival and weight gain, even if there was no difference in whole-udder milk yield between ewes with normal or defective whole-udders.

Udder halves that expressed very little or no milk during a milking event were excluded from somatic cell count (SCC) and milk composition analysis due to insufficient samples for laboratory testing. Hence, inference of SCC and milk composition parameters should be made in consideration of these limitations. The SCC cut-off point to indicate mammary infection in ewes has been reported to range from 300,000 to 1,000,000 [46], indicating a lack of consensus [47]. The present study found milk SCC was not different between defective and normal categories, as some individual udder halves in both categories were observed to have high SCC (i.e., more than one million). High SCC in the normal udders in the present study could be due to subclinical mastitis, while udder halves with lump (assumed to be abscesses) may change in bacterial phenotypic diversity in the mammary gland [26,48], which may result in variation in SCC.

In the current study, only milk solid non-fat (SNF) had any association with udder-half defects while there were no associations with crude protein, lactose, and total solids. There appears to be no available literature on the effects of palpable udder defects on milk composition in ewes. In a review, Martí-De Olives, et al. [27] reported that the concentration of lactose decreases and whey protein increases with subclinical mastitis while other milk components have been inconsistently influenced (decrease, increase, or no difference) by subclinical mastitis in ewes. Changes in fat and protein (casein) percentages depend on the extent of milk yield loss, due to the dilution or concentration effect. In this study, defective udder halves produced less milk than normal udder halves and the resulting concentration effect on milk composition may explain the lack of variation between defective and normal udder halves [7]. In addition, the total crude protein depends on the casein:protein ratio, which usually decreases as a result of infection [11,49,50]. However, whey proteins increase during mammary inflammation or damage to the blood milk-barrier, which is likely to leak from blood to milk [11,49]. Moreover, variation in milk composition due to mastitis depends on the type of infectious agents [7,51]. Generally, previous studies have summarized that quantitative milk production is a better indicator of early lamb growth than milk components; however, milk composition may explain a reasonable proportion [52,53].

## 5. Conclusions

In conclusion, the results of this study showed a high occurrence of palpable udder defects in early lactation in non-dairy Romney ewes with a previous history of udder defects. Further, palpable udder defects were associated with both udder-half and whole-udder changes in milk yield, in which the degree of milk loss depended on the type and persistence of the udder defects. Therefore, these findings indicate the importance of udder health in non-dairy ewes and the potential effect on their offspring. However, milk SCC and composition parameters, except SNF, presented no association with palpable udder defects. A high proportion of udder halves were hard seven days after lambing and expressed little to no milk but then changed to lump or normal over the following five weeks. The cause and pathogenesis of this defect is worthy of further consideration.

## Figures and Tables

**Table 1:** Description of udder-half and whole-udder analysis types (*n* = 12) and categories used to investigate the effects of palpable udder defects on milk parameters in mixed-age non-dairy breed (Romney) ewes, which were examined six times at weekly intervals, starting at day 7 of lactation.

Analysis Type and Number	Categories for Analysis	Description of Categories	Parameters Analysed
Weekly udder-half defect occurrence ^a^ (1)	Defective Normal (defective) Normal (normal)	Defective: udder-half categorised as hard or lump Normal (defective): normal udder-half but with hard or lump on the contralateral side Normal (normal): normal udder-half with a normal udder-half on the contralateral side	Udder-half milk yield
Overall udder-half defect occurrence (across all six examinations) (2)	Defective Normal (defective) Normal (normal)	Defective: udder-half categorised as hard or lump on at least one of the six occasions Normal (defective): normal udder-half contralateral side to overall defective Normal (normal): normal udder-half with a normal udder-half on the contralateral side in all six occasions	Udder-half milk yield, SCC, milk composition
Udder-half defect type at single point in time (weekly) (3)	Hard Lump Normal (defective) Normal (normal)	Hard: hardness in udder-half Lump: lump in udder-half Normal (defective): normal udder-half but with hard or lump on the contralateral side Normal (normal): normal udder-half with a normal udder-half on the contralateral side	Udder-half milk yield
Udder-half defect type change (overall) (4)	Hard-normal Lump-normal Hard-lump Hard-lump-normal Normal (defective) Normal (normal)	Hard-normal: udder-half was hard but subsequently changed to normal and remained normal Lump-normal: udder-half was lump but subsequently changed to normal and remained normal Hard-lump: udder-half was hard but subsequently changed to lump and remained as lump Hard-lump-normal: udder-half was hard but subsequently changed to lump and then changed to normal and remained as normal Normal (defective): normal udder-half contralateral side to overall defective Normal (normal): normal udder-half with a normal udder-half on the contralateral side in all six occasions	Udder-half milk yield
Udder-half defect frequency (5)	High Low Normal (defective) Normal (normal)	High: hard or lump identified on at least 3 examinations Low: hard or lump identified on 1 or 2 examinations Normal (defective): normal udder-half but with hard or lump on the contralateral side Normal (normal): normal udder-half with a normal udder-half on the contralateral side	Udder-half milk yield
Udder-half defect first time detection (6)	Day 7 Day 14 Normal (defective) Normal (normal)	Day 7: hard or lump first detected at first udder examination (Day 7) Day 14: hard or lump first detected at second or subsequent udder examinations (Day 14 or greater) Normal (defective): normal udder-half but with hard or lump on the contralateral side Normal (normal): normal udder-half with a normal udder-half on the contralateral side	Udder-half milk yield
Weekly whole-udder defect occurrence ^a^ (7)	Defective Normal (both)	Defective: One or both udder halves categorised as hard or lump Normal (both): both udder halves normal in all six occasions	Whole-udder milk yield
Whole-udder defect across all six examinations (overall) (8)	Defective Normal (both)	Defective: One or both udder halves categorised as hard or lump on at least one of the six occasions Normal (both): both udder halves normal in all six occasions	Whole-udder milk yield
Whole-udder defect type at each examination (weekly) (9)	Hard Lump Normal (both)	Hard: One or both udder halves categorised as hard or ‘lump’ on one udder-half and ‘hard’ on the other half Lump: One or both udder halves categorised as lump Normal (both): both udder halves normal	Whole-udder milk yield
Unilateral udder defect type change (overall) (10) ^b^	Hard-normal Hard-lump Hard-lump-normal Lump-normal Normal (both)	Hard-normal: one udder-half was hard but subsequently changed to normal and remained normal Hard-lump: one udder-half was hard but subsequently changed to lump and remained as lump Hard-lump-normal: udder-half was hard but subsequently changed to lump and then changed to normal and remained as normal Lump-normal: udder-half was lump but subsequently changed to normal and remained normal Normal (both): both udder halves normal in all six occasions	Whole-udder milk yield
Whole-udder defect frequency ^c^ (11)	High Low Normal (both)	High: hard or lump identified on at least 3 examinations Low: hard or lump identified on 1 or 2 examinations Normal (both): both udder halves normal	Whole-udder milk yield
Whole-udder defect first time detection (12)	Day 7 Day 14 Normal (both)	Day 7: One or both udder halves categorised as hard or lump first detected at first udder examination (Day 7) Day 14: One or both udder halves categorised as hard or lump first detected at second or subsequent udder examinations (Day 14 or greater) Normal (both): both udder halves normal	Whole-udder milk yield

Note: ^a^ Individual udder-half or whole-udder defects could change in their defect category over time. ^b^ Only unilateral udder defects were included (*n* = 170) to show change of defect over time. ^c^ Bilateral udder defect from a ewe on one observation was counted as a single occurrence.

**Table 2:** The effect of weekly ^4^ and overall ^5^ udder-half defect occurrence on udder-half milk yield (*n* = 352), somatic cell count (SCC) (*n* = 255), and milk composition (*n* = 343) (LSM + SEM) in non-dairy breed (Romney) ewes examined and milked once weekly during the first six weeks of lactation.

Dependent Variables	Udder-Half Defect Category					
	Normal (Normal) ^1^		Defective ^2^		Normal (Defective) ^3^	
		SEM	LSM	SEM	LSM	SEM
Udder-half milk yield	LSM					
Weekly defect ^4^ (g/d)	1118 ^b^	54.1	320 ^a^	83.7	1785 ^c^	90.4
Overall defect ^5^ (g/d)	1204 ^b^	76.8	507 ^a^	53	1811 ^c^	73.4
SCC (1000)	647	86.5	680	82.6	633	74.9
Milk composition						
Fat (%)	7.57	0.23	7.92	0.25	7.26	0.22
Crude protein (%)	4.95	0.04	4.83	0.04	4.89	0.04
Lactose (%)	7.57	0.23	7.92	0.25	7.26	0.22
Total solids (%)	18.3	0.25	18.7	0.29	18.5	0.26
SNF (%)	11.3 ^b^	0.07	11.0 ^a^	0.08	11.3 ^b^	0.07

^1^ Normal (normal): a normal udder-half where the contralateral udder-half was also normal. ^2^ Defective: udder-half categorised as hard or lump either in one or both udder halves. ^3^ Normal (defective): a normal udder-half where the contralateral udder-half was defective. ^4^ Weekly defect: udder-half defect category at each examination. ^5^ Overall defect: udder-half category for all six examinations (identified as defective by being ‘hard’ or ‘lump’ at least once during the six examinations). For weekly udder defect, individual udder-half could be identified in a different defect category each week whereas, the overall udder defect reflects a single overall udder-half category over the six-week (42 days) period. log-SCC: Natural log of somatic cell count. LSM: least square means. Different superscripts a,b,c within a row indicates significant differences (*p* < 0.05).

**Table 3:** Multivariate regression coefficient (Estimate ± SEM) of udder-half log-SCC (*n* = 255) and milk composition (fat, crude protein, lactose, total solids, and SNF) (*n* = 243) based on overall ^4^ udder-half defect from 30 non-dairy breed (Romney) ewes examined and milked weekly during the first six weeks of lactation.

Parameters	Intercept		Defective ^1^		Normal		Days in		SCC (10^6^)		Birth Rank		Rearing Rank		Defect History		R^2^
					(Defective) ^2^		Lactation										
	Est.	SE	Est.	SE	Est.	SE	Est.	SE	Est.	SE	Est.	SE	Est.	SE	Est.	SE	
Milk yield																	
Weekly ^3^	744.5	153.7	−739.40	98.9	755.6	103.7							224.1		103.9		0.366
Overall ^4^	1195.25	129.19	−762.73	153.78	674.07	168.13											0.386
Milk composition																	
Fat (%)	5. 97	0.32					0.04	0.01	0.71	0.19							0.67
Protein (%)	4.83	0.05							0.1	0.03							0.49
Lactose (%)	5.58	0.04							−0.19	0.04							0.32
Total solids (%)	17.05	0.34					0.37	0.05									0.64
SNF (%)	11.74	0.16	−0.30	0.18	0.07	0.2	−0.01	0	−0.26	0.06							0.3
Log-SCC	4.84	0.35									0.59	0.2			0.46	0.19	0.46

Normal (normal): a normal udder-half where the contralateral udder-half was also normal. Normal (normal) was used as a reference in all cases. ^1^ Defective: an udder-half categorised as hard or lump either in one or both udder halves. ^2^ Normal (defective): a normal udder-half but with hard or lump on the contralateral side. ^3^ Weekly defect: udder-half defect category at each examination. ^4^ Overall defect: udder-half category for all 6 examinations (identified as defective by being ‘hard’ or ‘lump’ at least once during the six examinations). For weekly udder defect, individual udder-half could be identified in a different defect category each week whereas, the overall udder defect reflects a single overall udder-half category over the six-week (42 days) period. Log-SCC: Natural log of somatic cell count. All intercepts and estimates were significantly different (*p* < 0.05), except where blank values are shown. Est.: indicates estimate of multivariate coefficient.

**Table 4:** Multivariate analysis coefficient (Estimate ± SEM) of weekly udder-half milk yield (g/d) based on either weekly ^6^ or overall ^7^ udder-half defect types from 352 individual udder-half observations from 30 non-dairy breed (Romney) ewes examined and milked weekly during the first six weeks of lactation.

Udder-Half Defect Parameters	Udder-Half Milk Yield (g/d)				*p*-Value
	Weekly Defect ^6^		Overall Defect ^7^		
	Estimate	SEM	Estimate	SEM	
Intercept	1255.49	151.37	1197.73	135.05	<0.001
Hard	−924.50	245.97			<0.001
Lump	−362.50	279.55			NS
Days in lactation	−71.17	32.86			0.03
Hard X days in lactation	229.74	75.85			<0.001
Lump X days in lactation	−2.82	67.20			NS
Normal (defective) X days in lactation	−152.43	60.07			0.01
Normal(defective) ^1^	943.35	215.62	647.77	170.15	<0.001
Hard-normal ^2^			−231.77	216.39	NS
Hard-Lump ^3^			−1303.20	195.39	<0.001
Hard-lump-normal ^4^			−1189.52	209.03	<0.001
Lump-normal ^5^			−356.22	192.04	NS
R^2^		0.28		0.53	

Normal (normal): normal udder-half with a normal udder-half on the contralateral side. Normal (normal) was used as a reference in all cases. ^1^ Normal (defective): normal udder-half but with hard or lump on the contralateral side. ^2^ Hard-normal: udder-half was hard but subsequently changed to normal and remained normal. ^3^ Hard-lump: udder-half was hard but subsequently changed to lump and remained as lump. ^4^ Hard-lump-normal: udder-half was hard but subsequently changed to lump and then changed to normal and remained as normal. ^5^ Lump-normal: udder-half was lump but subsequently changed to normal and remained normal. ^6^ Weekly defect: udder-half defect category at each examination. ^7^ Overall defect: udder-half category for all six examinations (identified as defective by being ‘hard’ or ‘lump’ at least once during the six examinations). For weekly udder defect, individual udder-half could be identified in a different defect category each week whereas, the overall udder defect reflects a single overall udder-half category over the six-week (42 days) period. All intercepts and estimates were significantly different (*p* < 0.05), except where blank values are shown.

**Table 5:** Multivariate analysis (estimate ±SE) of weekly udder-half milk yield (g/d) based on udder-half defect detection frequency and first defect detection time from 352 udder-half observations from 30 non-dairy breed (Romney) ewes examined and milked weekly during the first six weeks of lactation.

Parameters	Udder-Half Milk Yield (g/d)		R^2^
	Estimate	SE	
Effect of udder-half defect frequency			
Intercept (g/d)	1246.19	116.69	0.46
High (3–6) ^1^	−1081.73	149.02	
Low (1–2) ^2^	−266.16 ^NS^	156.67	
Normal (defective) ^3^	510.15	152.88	
Effect of udder-half defect first detection time			
Intercept	1279.23	115.67	0.29
Day 7 ^4^	−841.39	143.35	
Day 14 and above ^5^	−585.27	161.63	
Normal (defective) ^3^	526.49	163.68	

Normal (normal): normal udder-half with a normal udder-half on the contralateral side. Normal (normal) was used as a reference in all cases. ^1^ High: hard or lump identified on at least three examinations. ^2^ Low: hard or lump identified on one or two examinations. ^3^ Normal (defective): normal udder-half but with hard or lump on the contralateral side. ^4^ Day 7: hard or lump first detected at first udder examination at end of week one (Day seven). ^5^ Day 14: hard or lump first detected at second or subsequent udder examinations (Day 14 or greater). LSM: least square means. All estimates were significant (*p* < 0.05), except with NS.

**Table 6:** Multivariate analysis coefficient (estimate + SEM) of weekly whole-udder milk yield (g/d) based on either weekly^1^ or overall^2^ udder-half defect types from 170 individual whole-udder observations from 30 non-dairy breed (Romney) ewes examined and milked weekly during the first six weeks of lactation.

Dependent Variables	Category	Milk Yield (g/d)		R^2^
		Estimate	SEM	
Weekly whole-udder defect type ^1^				
Intercept		1532.8	255.7	0.26
Rearing rank		410.8	185.8	
Overall whole-udder defect type ^2^				
Intercept		1761.2	317.1	
Overall whole-udder defect type	Hard-normal ^3^	−232.1 ^NS^	238.3	0.29
	Hard-lump ^4^	−562.4	239.0	
	Hard-lump-normal ^5^	213.2 ^NS^	236.7	
	Lump-normal ^6^	−270.4 ^NS^	240.5	
Rearing rank		357.0	176.6	

^1^ Weekly whole-udder defect type: udder defect type at each examination. ^2^ Overall whole-udder defect type: unilateral defect type change across all six examinations. ^3^ Hard-normal: udder-half was hard but subsequently changed to normal and remained normal. ^4^ Hard-lump: udder-half was hard but subsequently changed to lump and remained as lump. ^5^ Hard-lump-normal: udder-half was hard but subsequently changed to lump and then changed to normal and remained as normal. ^6^ Lump-normal: udder-half was lump but subsequently changed to normal and remained normal. Normal: both udder halves normal (Reference). Normal (both) was used as a reference in whole-udder analysis. Note: Four ewes were not milked on day 42 (end of week six). NS: Not significant (*p* < 0.05).

## Data Availability

The data utilised by this study are available on request from the corresponding author.

## Appendix A

**Table A1:** Milkoscan FT 1 machine sheep milk calibrations for fat, crude protein, lactose, and total solids.

Component	Intercept	Coefficients	R-Squared	Absolute Accuracy	Relative Accuracy (%)
Fat (*N* = 51)	0.901	0.902	0.965	0.298	3.87
Crude protein (*N* = 54)	−0.61	1.11	0.977	0.067	1.134
Lactose (*N* = 51)	0.840	0.765	0.796	0.10	2.18
Total solids (*N* = 51)	1.878	0.909	0.986	0.206	1.113
SNF (*N* = 51)	−1.78	1.14	0.816	0.148	1.37

**Note:** The absolute accuracy expresses Standard Error of Prediction whereas the relative accuracy states Standard Error relative to the mean of the reference values.
